# The emerging roles of autophagy in intestinal epithelial cells and its links to inflammatory bowel disease

**DOI:** 10.1042/BST20221300

**Published:** 2023-04-13

**Authors:** Sharon Tran, Juliani Juliani, W. Douglas Fairlie, Erinna F. Lee

**Affiliations:** 1 Olivia Newton-John Cancer Research Institute, Heidelberg, Victoria 3084, Australia; 2 School of Cancer Medicine, La Trobe University, Bundoora, Victoria 3086, Australia; 3 Department of Biochemistry and Chemistry, School of Agriculture, Biomedicine and Environment, La Trobe Institute for Molecular Science, La Trobe University, Melbourne, Victoria 3086, Australia

**Keywords:** autophagy, Crohns disease, disease, gastrointestinal physiology, homeostasis, molecular basis of health and disease

## Abstract

Landmark genome-wide association studies (GWAS) identified that mutations in autophagy genes correlated with inflammatory bowel disease (IBD), a heterogenous disease characterised by prolonged inflammation of the gastrointestinal tract, that can reduce a person's quality of life. Autophagy, the delivery of intracellular components to the lysosome for degradation, is a critical cellular housekeeping process that removes damaged proteins and turns over organelles, recycling their amino acids and other constituents to supply cells with energy and necessary building blocks. This occurs under both basal and challenging conditions such as nutrient deprivation. An understanding of the relationship between autophagy, intestinal health and IBD aetiology has improved over time, with autophagy having a verified role in the intestinal epithelium and immune cells. Here, we discuss research that has led to an understanding that autophagy genes, including *ATG16L*, *ATG5*, *ATG7*, *IRGM*, and Class III PI3K complex members, contribute to innate immune defence in intestinal epithelial cells (IECs) via selective autophagy of bacteria (xenophagy), how autophagy contributes to the regulation of the intestinal barrier via cell junctional proteins, and the critical role of autophagy genes in intestinal epithelial secretory subpopulations, namely Paneth and goblet cells. We also discuss how intestinal stem cells can utilise autophagy. Importantly, mouse studies have provided evidence that autophagy deregulation has serious physiological consequences including IEC death and intestinal inflammation. Thus, autophagy is now established as a key regulator of intestinal homeostasis. Further research into how its cytoprotective mechanisms can prevent intestinal inflammation may provide insights into the effective management of IBD.

## Introduction

Inflammatory bowel disease (IBD) is considered a modern disease, having only emerged in the last 150 years [1] and being of higher prevalence in socio-economically advantaged populations [2]. Studies show that prenatal or perinatal exposure to antibiotics, use of oral contraceptives, or a Westernised diet of highly processed foods and high sugar content, can correlate with IBD [3–6]. These are factors that may explain how IBD disproportionately affects individuals in urbanised regions, although whether these are causative of IBD is yet to be proven unequivocally [3–6]. Approximately 6.8 million cases of IBD were reported across the globe in 2017 [2]. There are two clinically distinct classes of IBD — ulcerative colitis (UC) and Crohn's disease (CD) — although 5–15% of cases are classed as ‘indeterminate colitis’ [7]. The current paradigm for IBD aetiology is that individuals with genetic predispositions are exposed to triggering substances, for instance, bacteria or viruses, resulting in unresolved chronic inflammatory conditions driven by deregulated immune activity. Treatment options for IBD thus include an arsenal of both broad-acting and targeted immunosuppressive or immune-modulatory drugs that act to reduce inflammation. Still, the precise mechanisms of IBD aetiology remain unclear, and complete remission rates are generally reported to be below 50% [8–10].

In 2007, genome-wide association studies (GWAS) uncovered the core autophagy gene *ATG16L* to be a CD susceptibility locus [11–14]. Similarly, polymorphisms in other autophagy genes and autophagy-associated genes have been shown to be IBD-associated, including in *MTMR3* and *GPR65* [15,16], and also in *ULK1*, *IRGM, NDP52* and *PTPN2* for CD in particular [14,17–20]. This discovery of a link between *ATG16L* and CD catalysed research into the role of autophagy in the intestine and IBD. Here, we provide an updated review of the concepts that have emerged from this research, specifically the mechanisms and functions of autophagy proteins in intestinal epithelial cells (IECs) and intestinal stem cells (ISCs) and how these contribute to intestinal homeostasis and pathophysiology. We refer readers to other excellent reviews on how autophagy in immune cells contributes to IBD [21,22].

## Molecular features of autophagy

**Figure 1.**
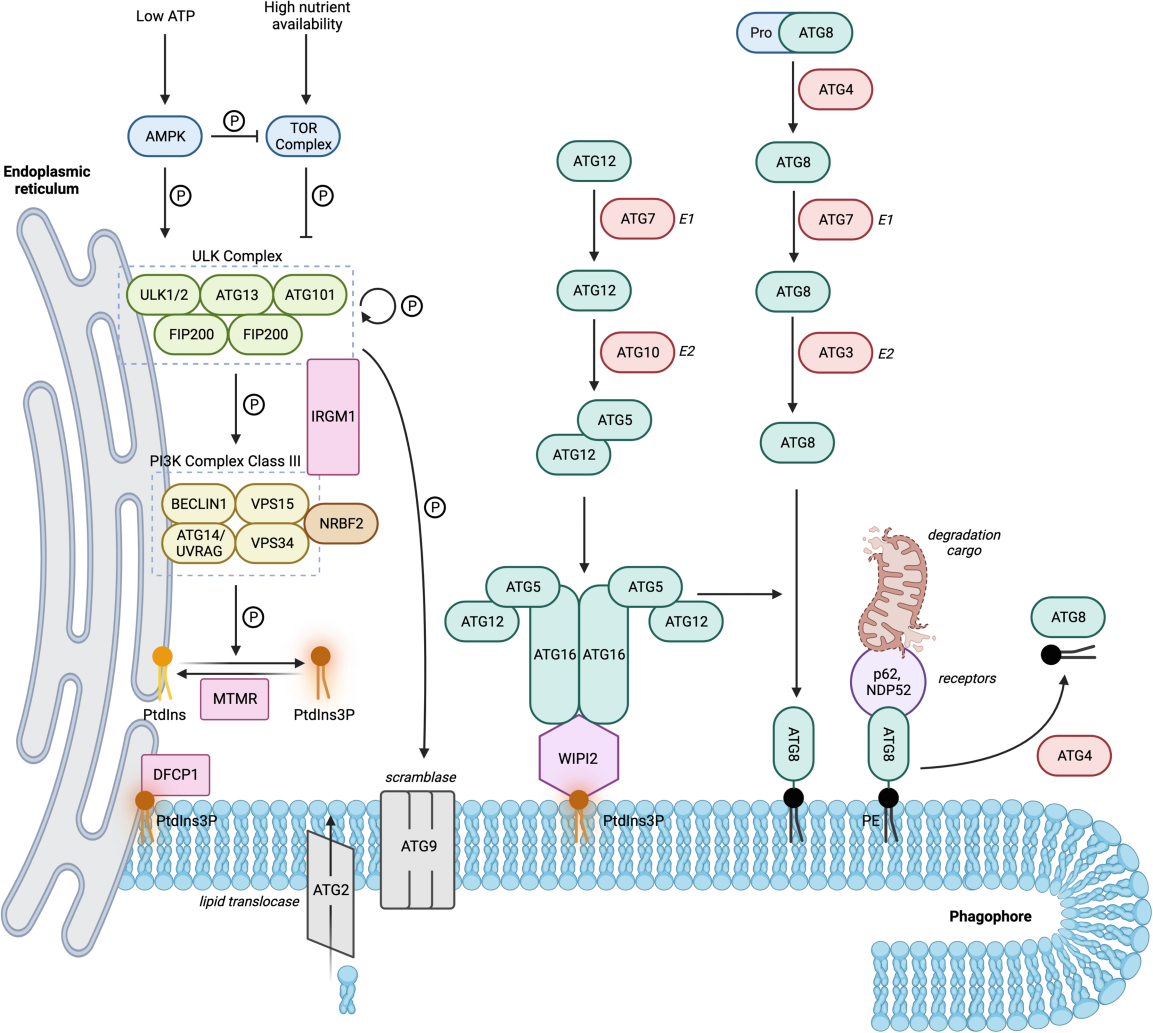
Schematic of the autophagy molecular network showing the major complexes involved in autophagosome initiation (ULK and PI3K Class III) and the two ubiquitin-like conjugation systems (ATG12–ATG5–ATG16L, and ATG8 lipidation) required for autophagosome expansion and capture of cargo.

Autophagy is defined as the lysosomal degradation of intracellular entities. This pathway can selectively degrade a diverse range of cargo, for example, protein aggregates or entire organelles such as mitochondria. Alternatively, non-specific targets can be degraded in bulk [23]. Three types of autophagy have been described based on the route of cargo delivery to the lysosome. Whilst chaperone-mediated autophagy (CMA) and microautophagy involve direct translocation through, or invagination of, the lysosomal membrane, respectively, macroautophagy (hereafter referred to as autophagy) is arguably more complex as it involves the formation of a *de novo* double membrane that encloses cargo. Cargo-enclosed vesicles can then mature and fuse with the lysosome. The overall pathway is summarised in Figure 1. The full names of all proteins are provided in the Abbreviations section.

### Autophagy initiation

Low energy or nutrient status of cells may be detected by AMPK to initiate autophagy via phosphorylation at distinct sites on ULK1. Conversely, the phosphorylation of ULK1 by TOR at a different site can restrict autophagy under high nutrient conditions [24]. Initiation of autophagy drives the serine/threonine kinase activity of ULK1/2, which is stabilised as a multimeric complex consisting of ULK1/2, FIP200, ATG101, and ATG13 to phosphorylate itself, ATG9 and members of the Class III PI3K complex [25–33]. The Class III PI3K complex, consisting of the lipid kinase VPS34, and subunits BECLIN1, VPS15 and either ATG14 or UVRAG, is then activated to convert phosphatidylinositol (PtdIns) to PtdIns3P, an important lipid constituent of autophagosome double membranes that recruits downstream effectors such as the tether WIPI2, and DFCP1 [34–41]. ATG2 has recently been uncovered to be a lipid translocase [42–44], whilst ATG9 is a transmembrane protein located on various vesicular compartments and described to have scramblase activity [45]. Together, ATG2 and ATG9 traffic lipids to supply the growing autophagosome membrane [42–44].

### Autophagosome expansion

Cargo sequestration and membrane expansion are supported by two ubiquitin-like conjugation systems that are localised to the autophagosome nucleation site by the ULK complex [46–49] and WIPI2 [40,50–53]. Firstly, the E1-like enzyme ATG7 activates and transfers ATG12 to the E2-like protein ATG10. This catalyses the conjugation of ATG12 to ATG5. ATG16L then interacts with ATG5 and self-associates to enable the formation of a multimer consisting of two ATG12–ATG5:ATG16L dimers [54]. Secondly, ATG4 cleaves pro-ATG8 proteins (LC3 or GABARAP) which enables them to be activated by the E1-like enzyme ATG7, passed onto the E2-like ATG3, before finally being conjugated to phosphatidylethanolamine (PE) by the E3 activity of the ATG12–ATG5:ATG16L multimer [54]. Lipidated ATG8s are incorporated into the growing phagophore membrane and serve to bind cargo with the assistance of cargo receptors and adaptors such as p62 and NDP52 [55,56].

### Autophagosome maturation and fusion

Steps following the sealing of the autophagosome are considered part of the maturation process. This can include the removal of some (but not all) ATG proteins from the phagophore surface [57], the transport of autophagosomes to, and fusion with, late endosomes and/or lysosomes, and the acquisition of amphisome (autophagosome fused with endosome) and autophagolysosome acidity and degradative hydrolase activity [58]. The molecular machinery employed in the transport and fusion processes is shared with the endosomal trafficking network and includes SNAREs, tethers, adaptor proteins, and RAB proteins. Here, SNARES on the autophagosome (for example, STX17 and YTK6) form complexes with those on the late endosome or lysosome (for example, VAMP8 and STX7), typically using the SNARE SNAP29 as an intermediary [58]. Tethers (including the HOPs complex, PLEKHM1 and EPG5) are SNARE chaperones that interact with autophagosome membrane components (for instance PtdIns, GABARABs, WIPI proteins), to promote SNARE complexes formation, but can also be multifunctional, particularly as RAB7 modulators. RAB7, a late endosome and lysosomal marker which interacts with LC3, PtdIns, NRBF (a subsidiary of PI3K complex 1), and others, appears essential to autophagosome maturation through its GTP hydrolysis function mediated by a range of effectors [59,60], and is additionally involved with autophagosome positioning due to its interactions with the transport adaptor protein FYCO1 [61].

### Other players

Several other proteins that are not directly involved in the generation of autophagosome membranes or the capture of cargo but can regulate some of these ‘core’ proteins to enhance or inhibit autophagy have also been investigated. Myotubularin phosphatases (MTMRs) are lipid phosphatases that can dephosphorylate PtdIns3P (and other lipids), to either promote autophagosome formation or reduce autophagic flux [62]. IRGM is an interferon (IFN)-inducible protein with pro-autophagic activity through interactions with both ULK1 and BECLIN1 to promote the assembly of autophagy initiation complexes [63]. Interactions between BECLIN1 and other proteins, such as pro-survival BCL-2 family members or AMBRA1, inhibit or promote autophagy, respectively [64]. The PI3K complex also binds additional auxiliary proteins such as NRBF2, forming subcomplexes that can be pro-autophagic or autophagy-inhibiting [64]. LRRK2, which harbours both GTPase and RAB-targeting kinase activity, is associated with both autophagy and endosomal trafficking, with context-dependent repression or activation of autophagy [65,66]. A G protein-coupled receptor involved in cAMP and Rho signalling pathways, and proton sensing, GPR65, was shown to be important for controlling lysosomal pH and the degradative capacity of this organelle [16]. Finally, the tyrosine phosphatase PTPN2 is required for efficient autophagosome formation but the mechanisms are still poorly understood [67].

## Intestinal physiology

**Figure 2.**
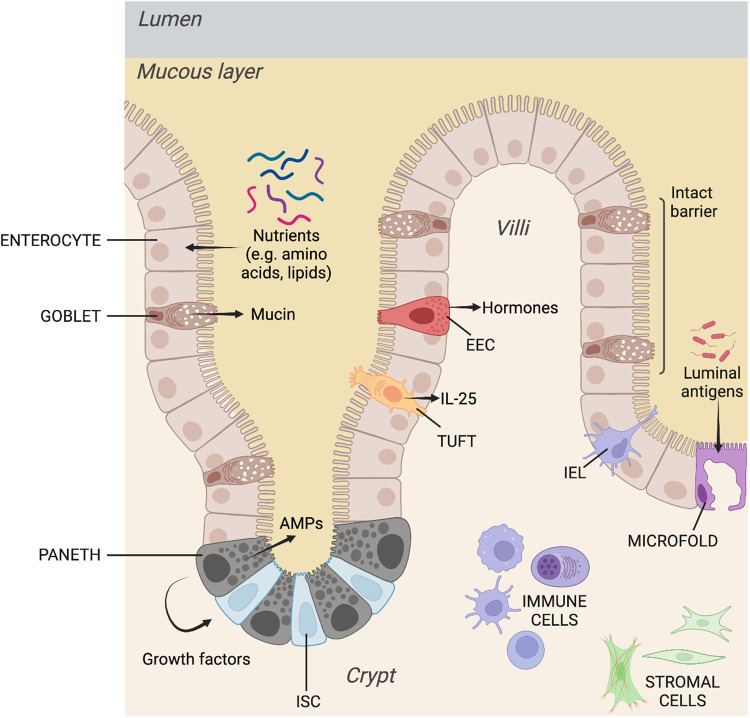
Anatomy and physiology of the small intestinal mucosa showing the major cell types and their relative locations within the repeating crypt-villi structures.

The small intestinal mucosa is organised into repeating crypt-villi structural units where a monolayer of polarised intestinal epithelial cells (IECs) reside over the stroma (Figure 2). Intestinal stem cells (ISCs) in the crypt divide and differentiate to give rise to the absorptive and secretory IEC lineages which perform different functional roles. Enterocytes are the most abundant IEC subtype and carry out digestive and nutrient-absorptive functions. Microfold (M) cells reside over intestinal lymphoid patches and sample the intestinal lumen, thus regulating immunotolerance to luminal antigens.

There are four secretory IEC subpopulations. Paneth cells reside in the crypt interspersed between ISCs and secrete antimicrobial peptides (AMPs) such as lysozyme, as well as growth factors to support the stem cell niche. Goblet cells are critical for generating mucins, which form the principal component of the protective physicochemical mucous barrier between the lumen and IECs. Enteroendocrine cells (EECs) secrete hormones in response to luminal conditions, contributing to gut mobility, nutrient absorption, satiety, and other effects. Finally, tuft cells have a chemosensory role and can secrete IL-25 to initiate type 2 innate immune responses, ultimately resulting in the expulsion of invasive helminths and protozoan parasites from the intestinal lumen [68].

Intestinal barrier permeability is also a key factor determining intestinal homeostasis and affects the development, pathogenicity and severity of IBD [69]. Maintenance of this barrier is controlled largely by intercellular junctional complexes (and their associated transmembrane proteins). This consists of adherens junctions (catenins, cadherins), tight junctions (ZO-1, claudin-2, occludins, Junctional Adhesion Molecules), and desmosomes (desmoglein, desmocollins, etc.). Junctional complexes regulate intercellular contact between adjacent IECs, sealing the intercellular space to protect the host from potentially harmful luminal agents, whilst being selectively permeable to water, nutrients, and electrolytes [70,71].

Structural support for IECs is provided by the underlying stromal cells that can also secrete factors to regulate IECs in a paracrine fashion. The relationship between IECs and the stroma is also bi-directional, where IECs can also, in turn, regulate stromal activity. Immune cells are also present in the mucosa as intraepithelial lymphocytes (IELs) residing between adjacent IECs, as well as the presence of both the myeloid and lymphoid immune compartments in the stroma. These three branches, IECs, stroma and immune cell-mediated effects, together with the microbiome, form a system that enables the intestine to perform its digestive and absorptive functions whilst maintaining intestinal barrier integrity and immunity against the pathogenic or noxious agents they might be exposed to in the lumen.

While the symptoms of UC and CD are similar (bloody stool, weight loss, abdominal pain), the diseases are characterised by several different features. UC is localised to the colon and rectum, and inflammation is restrained to the mucosal layer, resulting in an ulcerated appearance of the intestinal wall with complications that can include toxic megacolon. The UC inflammatory cytokine signature consists of those involved in the immune pathways for T_h_2 (IL-5, IL-6, IL-13, IL-33 and TNF), T_h_9 (IL-9, IL-33), and T_h_17 (IL-1β, IL-6, IL-17, IL-23, (tumour necrosis factor) TNF) response [72]. In contrast, CD can affect any part of the gastrointestinal tract from the mouth to the rectum in a non-contiguous manner. Inflammation is transmural and can result in the intestinal wall having a ‘cobblestone' appearance with fistulas as likely complications. CD is considered to be characterised to have a T_h_1 (IL-6, IL-12, IL-18, INFγ, TNF) as well as a T_h_17 response [72]. Microbiota appears to have less diversity for both UC and CD patients compared with healthy subjects, although dysbiosis is reportedly greater in CD than UC [73–75]. Different genetic risk variants and likely further heterogeneity even within these two IBD entities [76] underscore aetiological differences that may drive the development of these different features. Interestingly, from GWAS alone, autophagy gene polymorphisms appear to be more strongly linked to CD.

## Xenophagy in IECs is an innate defence mechanism against pathogens and pathobionts

**Figure 3.**
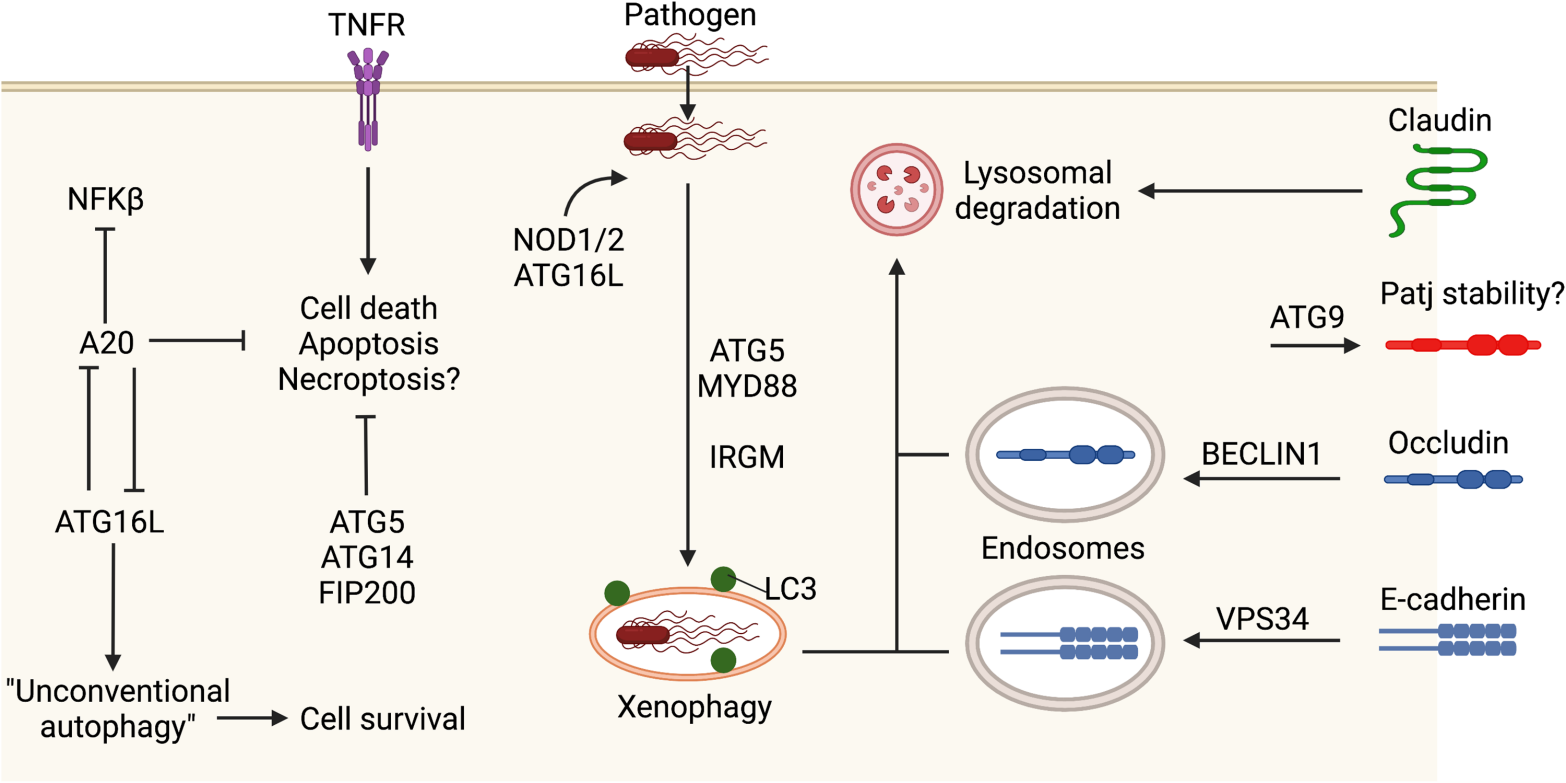
Autophagy genes and cellular processes such as intestinal epithelial barrier function and protection from cell death associated with xenophagy in response to pathogens.

Perhaps the most straightforward mechanism by which autophagy protects the intestinal epithelium is through xenophagy in IECs, or the autophagic degradation of bacteria that have invaded IECs. This can be visualised by the colocalisation of LC3 puncta with bacteria, which can become diminished when autophagy proteins are compromised. Expression of mutant *ATG16L* harbouring the CD-associated risk polymorphism, *ATG16L^T300A^,* in the Caco2 colorectal carcinoma line shows that these cells have impaired capture of *Salmonella typhimurium* in LC3-positive autophagosomes [77], a bacteria that causes gastroenteritis and is associated with an increased risk of CD or UC [78]. Loss of *Atg5* in the intestinal epithelium was also associated with increased *S. typhimurium* burden in IECs and the extraintestinal dissemination of this pathogenic bacteria [79]. Similarly, the expression of *IRGM* (which also harbours CD risk variants) is associated with autophagic flux and attenuating the replication of the CD-associated adherent-invasive *Escherichia coli* (AIEC) in cell lines [80]. The capture of bacteria appears to utilise the intracellular sensors NOD1 and NOD2, which recruit ATG16L to the plasma membrane at the site of bacterial entry (Figure 3). Cells homozygous for a CD-associated *NOD2* frameshift mutation fail to recruit ATG16L to the plasma membrane and show impaired bacterial capture in autophagosomes [81]. The major signalling adaptor molecule of the toll-like-receptors (TLRs) family, MyD88, also appears essential in IECs for the xenophagy response to *S. typhimurium* (Figure 3). In mice deficient for MyD88, a challenge with *S. typhimurium* does not induce the formation of LC3 puncta [79]. Thus, carrying CD risk variants of autophagy genes that impair clearance of IBD-associated pathogens appears to be an underlying contributor to Crohn's development.

## ATG16L and the conjugation machinery maintain Paneth cells

**Figure 4.**
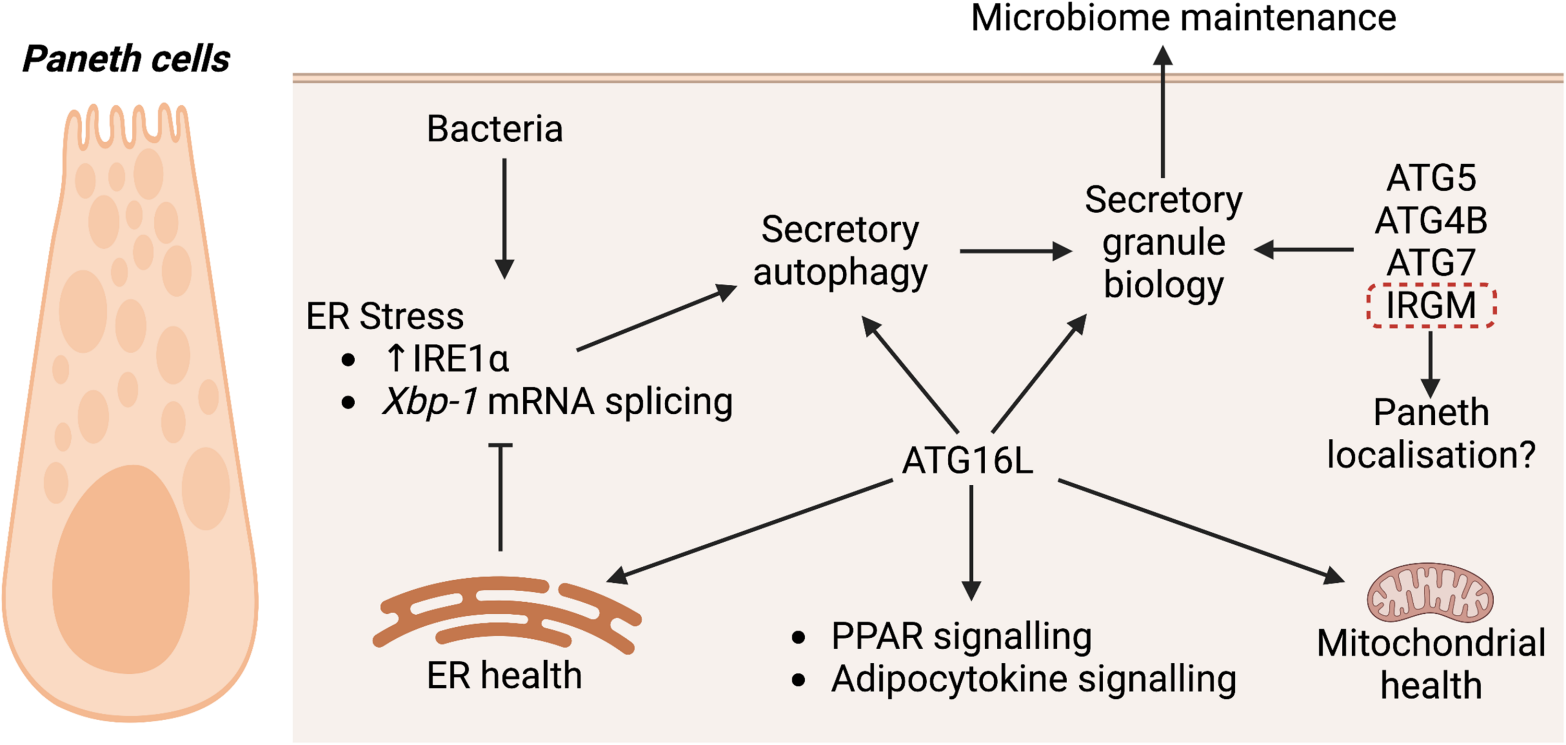
Autophagy genes and associated processes implicated in the maintenance of Paneth cells in response to bacterial infection.

As briefly described above, ATG16L forms part of the multimeric ATG12–ATG5:ATG16L complex that acts as an E3-like enzyme for ATG8 lipidation (Figure 1). The function of the ATG16L protein has been a major focus of studies on intestinal autophagy since its identification as a core autophagy candidate in GWAS studies for IBD. In addition to its role in xenophagy, evidence from multiple studies demonstrates that there is a clear role for ATG16L in maintaining Paneth cell secretory granules, which contain AMPs and immunomodulating proteins, and Paneth cell homeostasis (Figure 4). In mice with an IEC-specific knock-out of *Atg16L*, Paneth cell numbers were reduced [82], with diminution of both their morphology and size of secretory granules [83], and an accumulation of the endoplasmic reticulum (ER) stress sensor IRE1α [83,84]. Mice with a knock-in of hypomorphic *Atg16L* showed Paneth cells characterised by degenerating mitochondria and transcriptional up-regulation of the peroxisome proliferator-activated receptor (PPAR) and adipocytokine signalling pathways [85]. Generation of mice bearing a knock-in of the genetic equivalent to the human CD susceptibility allele *ATG16L*^T300A^ showed that Paneth cells also had reduced amounts of lysozyme and that these mice had an altered microbiota composition [86,87].

Similar phenotypes have been reported for genetic knock-out models of other autophagy conjugation proteins. In mice with IEC-specific *Atg5* loss, Paneth cells also had a diminished morphology, were reduced in numbers, and showed dissipating granules [85,88]. These mice were also associated with an altered microbiome [89]. Reduced Paneth granule size was also observed in IEC-specific *Atg7* knock-out mice [83,90], and reduced lysozyme staining was observed in the Paneth cells of whole-body *Atg4B*-deficient mice [91]. Each of these strains of mice with modified autophagy conjugation genes, namely *Atg16L, Atg5, Atg7* and *Atg4B*, showed worsened prognostic outcomes to chemical, physical or bacterial intestinal insults that resulted in the development of IBD-like pathologies. This included lower survival and poorer histological scores of the intestinal tract compared with control mice in response to the intestinal barrier-disrupting agent dextran sodium sulfate (DSS, which gives rise to a UC-like phenotype), the development of spontaneous age-dependent CD-like transmural ileitis, chronic colitis (indeterminate if UC or CD) in response to the opportunistic bacteria *Helicobacter hepaticus*, increased mortality from enteritis induced by *Toxoplasma gondii*, and poorer clinical disease scores against murine norovirus which results in an indeterminate, possibly transmural form of enterocolitis [79,82–93]. Notably, as the colon does not have any villi, Paneth cells are generally not found in the colon, although there are analogous Paneth-like cells [94]. This may provide an explanation for why autophagy genes do not exclusively protect the colon, but are protective of the larger gastrointestinal tract.

Combined, the data indicates that on a basal level, ATG16L in Paneth cells protects against ER stress, mitochondrial degeneration, regulates PPAR and adipocytokine signalling, and is important in the biogenesis of the secretory granules in these cells. However, the mechanistic pathways between ATG16L, other conjugation proteins, and these functional outcomes are still not well understood. In IECs, it has been shown that the WD40 domain of ATG16L interacts with the anti-inflammatory protein A20 [95]. This was proposed to be a mutually reciprocal relationship, where ATG16L promoted the lysosomal degradation of A20, and A20 promoted the ubiquitin-mediated degradation of ATG16L. Loss of both these proteins in the mouse intestinal epithelium resulted in spontaneous enterocolitis, marked by increased IL-1β and TNF and thickening of the jejunal wall, features suggestive of CD pathology. These were attributed to up-regulated NFκβ signalling and cell death (which is suppressed by A20) and a lack of ATG16L1 WD40 domain-mediated ‘unconventional' autophagy (Figure 3) [95].

The enhanced levels of intestinal pro-inflammatory cytokines as just described above is a common occurrence under challenging conditions where ATG16L or conjugation protein integrity are additionally compromised, even if these genetic modifications target the IEC compartment rather than immune cells. For instance, increased TNFα and IL-1β levels are observed following LPS-stimulated NFκβ signalling in IEC-specific ATG7-deficient mice [96]. Conjugation protein-deficient intestinal cells appear particularly further sensitised to death triggered by TNF. Intestinal organoids derived from IEC-specific ATG16-deficient mice showed increased necroptosis [82] or apoptosis [92] when treated with TNFα. Similarly, TNF-treated ATG5-deficient intestinal organoids had decreased viability [88]. Thus, conjugation proteins enable IEC survival under duress from inflammatory cytokines and a pathogenic or chemically insulting environment (Figure 3). This cell death likely contributes to, and/or further exacerbates, intestinal inflammatory symptoms.

During bacterial infection which induces ER stress, ATG16L is leveraged to help lysozyme secretion from Paneth cells in another unconventional autophagy process termed ‘secretory' autophagy (Figure 4) [97]. Loss of both *Atg16L1* and the ER stress/unfolded protein response (UPR)-mediator *Xbp-1* (also a risk locus for both UC and CD [98]) in the intestinal epithelium results in spontaneous severe transmural enteritis [83]. In a DSS-induced colitis model, stimulation of colonocytes with TNFα and NOD ligands promotes IKKα to phosphorylate ATG16L1 (Ser278), stabilising ATG16L1 against degradation, which is associated with protection against IREα-mediated ER stress and activation of caspase-12, which causes a loss of cytoprotective IL-18 [99]. Interestingly, the CD-associated ATG16L^T300A^ mutated protein is more susceptible to caspase-3 or caspase-7 mediated degradation resulting in diminished autophagy [86,100].

Although limited data currently exists, studies on IRGM suggest that non-conjugation autophagy proteins work differently from conjugation proteins in Paneth cells. Mice with a systemic deficiency of *Irgm1* have Paneth cells with a different morphology to *Atg16L*-deficient mice. These Paneth cells can be ectopically located further up the villi and appear swollen, with a smaller granule core and a halo that is electron lucent when viewed with transmission electron microscopy [101]. Mice have down-regulated expression of the Paneth antimicrobial genes *Lys* (lysozyme) and *Defa20* and are susceptible to both DSS-induced colitis and ileitis [101]. The role of IRGM1 has only more recently been elucidated and promotes ULK1 and BECLIN1 association to enhance autophagosome nucleation [63]. IRGM1 can also interact with NOD2 [63], and is reported to interact with the inflammasome NLRP3 and ASL proteins to prevent their oligomerisation, as well as with p62 to mediate p62-dependent selective autophagy of NLRP3 and ASL, thus suppressing IL-1β maturation, pyroptosis and protection against caspase-1 activity in a DSS-colitis model [102]. IRGM is associated with mitophagy [101] and has affinity for the mitochondrial lipid cardiolipin that promotes mitochondrial fission through autophagy [103]. The full pathway between these IRGM interactions and Paneth granules and other biology is yet to be elucidated.

## Autophagy maintains goblet cells and promotes their degranulation

Goblet cells are a second secretory cell type that depend on the autophagy machinery for their function. *Atg16L*^T300A^ mice show colonic and ileal goblet cells with increased mucin area and decreased mucin secretion [86,87]. Similarly, enlarged mucin granules were observed in colonic goblet cells of mice with an intestinal-specific deficiency of *Atg5* [104]. In colon epithelial conditional *Atg7* knock-out mice, decreased mucin expression and secretion were also observed, together with transcriptional decreases in several antimicrobial and antiparasitic peptides and microbiome changes [105]. Concordantly, *Becn1*^F121A^ knock-in mice, which express a mutant BECLIN1 with decreased BCL-2 binding that enhances its availability for autophagic flux, have a thicker colonic mucosal layer and reduced ER stress levels [106]. Conversely, *Bcl2*^AAA^ knock-in mice, which express a mutant BCL-2 that constitutively binds BECLIN1 and reduces its availability for autophagic flux, have a thinner mucosal layer in the colon [106]. As mentioned above, *Atg16L*^T300A^ mice and IEC-specific *Atg5* knock-out mice are sensitised to bacterial or DSS-induced forms of intestinal inflammation, and this is also reflected in *Bcl2*^AAA^ mice which have an altered microbiome and are sensitive to DSS or AIEC colitis [106].

**Figure 5.**
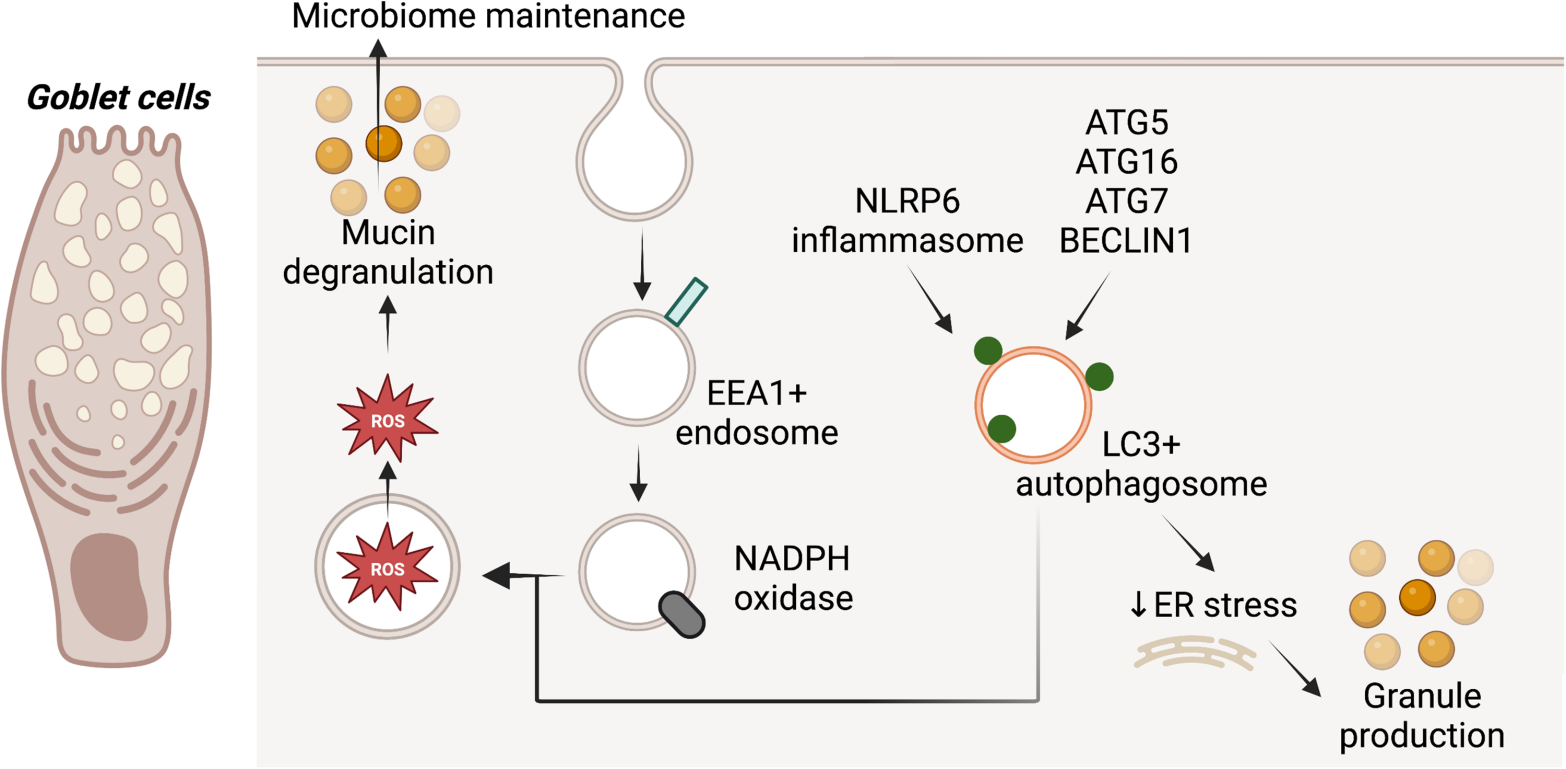
Autophagy genes are associated with goblet cell granule production and degranulation.

Common to both Paneth and goblet cells is the protection offered by autophagy against ER-stress that likely contributes to the proper generation of secretory granules, given the importance of the ER in protein production (Figure 5). Mucin-2 (MUC2) production was associated with high constitutive levels of autophagy in colonoids or goblet cell lines, and as such, autophagy was proposed to promote cell survival during production-associated metabolic stress [107]. In addition to granule generation, degranulation may also utilise autophagy proteins. Reactive oxygen species (ROS)-dependent degranulation in goblet cells was reported to require the convergence of LC3-, NADPH- and EEA1-containing compartments (Figure 5) [104]. Secretion also appears to depend on the NLRP signalling pathway-mediated induction of autophagy or formation of LC3-puncta (Figure 5) [108]. It is unclear if similar degranulation mechanisms are also employed by Paneth cells. It is also uncertain if small intestinal goblet cells are moderated in the same manner as colonic goblet cells.

## The regulation of ISCs by autophagy

**Figure 6.**
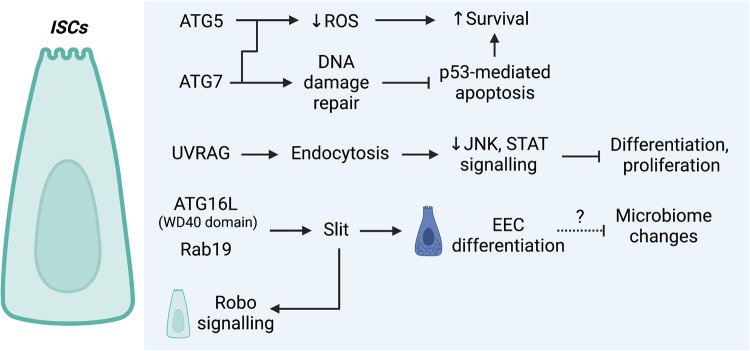
In ISCs, autophagy genes are associated with the regulation of ROS and DNA damage-associated stress, and through non-autophagy mechanisms that have been associated with differentiation.

Autophagy appears to be utilised by ISCs to moderate ROS and DNA damage-associated stress to ensure ISC survival (Figure 6). In mice with an intestinal-specific loss of *Atg5*, numbers of ISCs are reduced and surviving ISCs show increased ROS levels [93]. In mice with an ISC-specific loss of *Atg7*, a similar lack of ROS clearance was observed as well as inefficient DNA damage repair that was associated with p53-mediated apoptosis of ISCs [109]. Interestingly, an autophagy-independent role in ISC differentiation into EECs via signalling has been reported for ATG16L (Figure 6). It was found in drosophila that the WD40 domain of Atg16L is required to bind Rab19, and these proteins together maintain the production of the ligand Slit, which can activate Robo receptor-mediated signalling in ISCs. Loss of this signalling pathway resulted in a decline in the number of mature EECs and was associated with a spontaneous intestinal inflammatory signature in these flies [110]. Another autophagy-independent mechanism has also been reported for UVRAG in ISCs. A loss of drosophila UVRAG in ISCs results in an accumulation of endocytosed ligands and sustained activation of JNK and STAT signalling [111]. Animals suffer from impaired differentiation and uncontrolled proliferation associated with gut dysfunction and reduced lifespan [111]. As a colorectal cancer-associated tumour suppressor, this suggests a mechanism involving the deregulation of endocytic trafficking functions rather than autophagy functions of UVRAG as a mechanism of colorectal carcinoma development in humans.

## Autophagy genes regulate the intestinal barrier

It is emerging that autophagy has a crucial role in the regulation of the intestinal epithelial barrier, where a loss of autophagy gene expression can lead to alterations in the expression or distribution of intestinal junctional proteins [112–117]. Induction of autophagy via starvation reduces gut epithelial barrier permeability due to the increased lysosomal degradation of claudin-2 in Caco-2 cells (Figure 3) [112]. Similarly, ablation of Atg9 in drosophila results in the aberrant formation of the midgut, accompanied by dramatically enlarged enterocytes as well as barrier dysfunction and reduced fly lifespan. This was attributed to the loss of PALS1-associated tight junction protein (Patj), which is an Atg9-interacting protein (Figure 3) [113].

It is increasingly apparent that many autophagy proteins are multifunctional and that this has physiological consequences. More specifically, members of the Class III PI3K complexes have endosomal trafficking functions that can help maintain intestinal homeostasis through regulation of the intestinal barrier. Loss of *Vps34* in zebrafish resulted in disrupted barrier integrity due to defective E-cadherin trafficking to the cell surface (Figure 3), giving rise to epithelial injury, a malformed intestinal adhesion belt, inflammation, and premature death [115]. Additionally, BECLIN1 also plays an autophagy-independent role by associating with the tight junction protein, occludin, and regulating its constitutive endocytosis from the membrane (Figure 3), which subsequently modulates gut barrier permeability [116,117]. As described above, UVRAG in drosophila ISCs is also required for endosomal trafficking of cell surface receptors and ligands and leads to gut dysfunction and shortened lifespan when disrupted, though the impacts on barrier permeability were not directly investigated [111]. It is interesting that whilst ATG14 is a member of the Class III PI3K Complex 1 not typically associated with endosomal trafficking, there is some evidence it can play a role in this process [118]. Accordingly, ATG14 (and FIP200) intestinally deficient mice develop a spontaneous intestinal atrophy phenotype six weeks after birth due to severe loss of villi from an increase in intestinal epithelial apoptosis triggered by TNF [119]. This is unlike the knock-out of conjugation proteins in the intestinal epithelium, which have a milder, stimulus-induced intestinal inflammatory phenotype. This suggests that autophagy genes may have additional functions essential for the regulation of intestinal development and basal homeostasis.

## Targeting autophagy to treat IBD

Beyond surgery, approved treatment options for IBD generally act to suppress the inflammatory response associated with UC and CD. These include 5-aminosalicylic acid, corticosteroids and immunomodulators such as methotrexate, thiopurines and calcineurin inhibitors that act on a range of targets to suppress immune cell activation, migration and proliferation via transcriptional and other mechanisms that reduce the production of pro-inflammatory cytokines [120,121]. Such treatments are efficacious in UC and/or CD, however, most are associated with side effects that are often serious and patients can become refractory to treatment. More recently, more selective biologic therapies, including monoclonal antibodies targeting inflammatory cytokines TNF, interleukin IL-12 and IL-23 or leukocyte homing integrin α4β7, and small molecule therapies (e.g. Janus kinase inhibitors, sphingosine-1-phosphate receptor modulators) have been shown to be relatively effective in the management of IBD, however, a significant proportion of patients lose response to these advanced therapies over time [120].

Interestingly, some of the aforementioned immunosuppressive drugs can also modulate (either inhibiting or activating) autophagy, and this ‘secondary' activity may account for at least part of their mechanism of action [122]. Activities such as exercise and caloric restriction that can induce autophagy have shown benefits in IBD models and patients in some studies, however, it is unclear whether autophagy induction itself has any role in these outcomes [123,124]. Moreover, despite the clear involvement of deregulated autophagy in IBD, no treatments that directly target autophagy pathway components have yet to be approved for UC or CD. An early study investigated hydroxychloroquine, an inhibitor of lysosomal degradation, post-surgery in a small number of patients but, perhaps not surprisingly, was ineffective [125] given the more recent knowledge regarding the impact of defective autophagy in IBD. The obvious rationale that induction of autophagy may be therapeutically useful in treating IBD led to the testing of rapamycin (Sirolimus), an inhibitor of mTOR, in CD patients, and was shown to be effective in some cases [126,127]. However, it is unclear if these benefits are due to the known immunosuppressive effects of rapamycin or its induction of autophagy, though a recent study in an *IL-10* knock-out model of CD suggested it could decrease intestinal and colonic permeability through its pro-autophagy activity [128]. Otherwise, very few inducers of autophagy have been developed that are highly specific for the components of the autophagy machinery. One exception is the Tat-Beclin peptide derived from the BECLIN1 protein, although its mechanism is via interaction with autophagy regulator Golgi-associated plant pathogenesis related protein (GAPR-1) rather than with BECLIN1 itself [129]. Nevertheless, this peptide induces autophagy *in vivo* and is effective in disease models where autophagy is impaired (e.g. neurodegenerative disease) [129]. Compounds such as BH3-mimetics which target the BCL-2 proteins that negatively regulate BECLIN1, could also be a therapeutic option, although they may potentially activate apoptotic pathways [130]. Interestingly, a recent report suggested that BH3-mimetics can be developed that selectively target the BCL-2:BECLIN1 interaction over interactions with pro-apoptotic proteins [131], whilst another study showed cell-type selective induction of autophagy (albeit autophagic death) *versus* apoptosis in response to BH3-mimetics [132]. However, neither BH3-mimetics nor Tat-Beclin is yet to be investigated in IBD models. One ever-present consideration, however, is whether such an approach will work in the context of mutations in autophagy genes such as ATG16L where it may not be possible to (re-)activate the pathway. Such issues may eventually be negated through the use of gene editing approaches which are starting to be investigated in models of IBD [133,134], however, there are many significant hurdles and associated risks that would need to be surmounted before these techniques could be applied in patients.

## Concluding remarks

IBD is a multifactorial disease in which deregulated autophagy has been established as one of the possible factors contributing to disease aetiology. The physiological roles of autophagy in the intestine are diverse. It mediates IEC xenophagy, maintains the intestinal barrier (attributed at least in part to emerging roles of key core proteins in endosomal trafficking), and promotes cell survival. In Paneth and goblet cells, the presence of autophagy is required for proper secretory granule biogenesis and impacts granule release. Autophagy proteins are important for the regulation of ROS levels and even have unconventional roles in cell signalling that regulate proliferation and differentiation. Importantly, although not discussed in detail in this manuscript, autophagy regulates myeloid and lymphoid intestinal immune populations to regulate a plethora of functions including cytokine secretion, bacterial clearance, antigen processing, and cell survival, where a loss of autophagy protein integrity is also associated with susceptibility to DSS-induced colitis in mice. Little has been published on how autophagy proteins function in non-immune cells of the intestinal stroma, however recently, it was shown that ATG5 or FIP200 was essential in PDGFRα-positive mesenchymal stem cells in order to generate Wnt ligands necessary for maintaining the stem cell niche [135]. Loss of these proteins in these cells resulted in intestinal epithelial apoptosis, villi blunting and the rapid and fatal deterioration of mice [135], highlighting the critical importance of these proteins in this compartment. This is an area where further investigation is warranted. Similarly, research into the role of autophagy in tuft cells and M cells currently has not been reported. Given the known links between autophagy and secretion, and autophagy and antigen processing, it is likely that autophagy will play a role in the biology of these two cell types.

Despite its multifactorial pathophysiology, dual therapy with current treatments for IBD is relatively novel [136]. Although targeting deregulated autophagy, such as through anti-TNF, is considered effective at inducing and maintaining clinical remission, agents that directly promote autophagy have yet to be clinically tested as monotherapy or in combination with existing therapies in IBD. A greater understanding of the molecular pathways of autophagy in intestinal physiology may provide profound insight into innovative drug development and precision-based strategies in IBD.

## Perspectives

- Inflammatory bowel disease (IBD) is a complex disease that impacts millions of people globally. Promising treatments do exist, but patients frequently relapse and there is a poor understanding of the specific mechanisms in disease aetiology.
- Mutations in autophagy genes are associated with IBD and deregulated autophagy is strongly associated with the loss of intestinal homeostasis due to the role of autophagy proteins in IECs, immune cells and stromal cells.
- Multifunctional autophagy proteins have an emerging role in IEC health through their endosomal trafficking roles but have had little investigation so far. Elucidating how autophagy proteins contribute to the maintenance of intestinal cell subpopulations and intestinal physiology offers insights into how we can potentially treat IBD patients, particularly those harbouring autophagy gene mutations.

## Open Access

Open access for this article was enabled by the participation of La Trobe University in an all-inclusive *Read & Publish* agreement with Portland Press and the Biochemical Society under a transformative agreement with CAUL.

## Abbreviations

AIEC: adherent-invasive *Escherichia coli*
AMBRA1: activating molecule in *BECN1*-regulated autophagy protein 1
AMPK: AMP-activated protein kinase
AMPs: antimicrobial peptides
ATG: autophagy-related protein
BCL-2: B cell lymphoma 2
CD: Crohn's disease
CMA: chaperone-mediated autophagy
DFCP-1: double FYVE-containing protein-1
DSS: dextran sodium sulfate
EEA1: early endosome antigen 1
EEC: enteroendocrine cell
EPG5: ectopic P-granules 5 autophagy tethering factor
ER: endoplasmic reticulum
FIP200: focal adhesion kinase family interacting protein of 200 kDa
GABARAP: gamma-aminobutyric acid type A receptor-associated protein
GAPR-1: Golgi-associated plant pathogenesis related protein
GPR65: G protein-coupled receptor 65
GWAS: genome-wide association studies
HOPS: homotypic fusion and protein sorting
IBD: inflammatory bowel disease
IEC: intestinal epithelial cell
IEL: intra-epithelial lymphocyte
IFN: interferin
IRE1α: inositol-requiring enzyme 1α
IRGM: immunity-related GTPase family M protein
ISC: intestinal stem cell
LC3: microtubule-associated protein 1A/1B-light chain 3
LPS: lipopolysaccharide
LRRK: leucine-rich repeat serine/threonine-protein kinase 1
MTMR: myotubularin phosphatase
mTOR: mammalian target of rapamycin
MUC2 mucin 2: oligomeric mucus/gel-forming
NADPH: nicotinamide adenine dinucleotide phosphate
NDP52: nuclear domain 10 protein 52
NFκB: nuclear factor kappa B
NLRP: NOD-, LRR- and pyrin domain-containing protein
NOD: nucleotide-binding oligomerisation domain-containing protein
NRBF2: nuclear receptor binding factor 2
p62: sequestosome-1
PDGFRα: platelet-derived growth factor receptor α
PE: phosphatidylethanolamine
PI3K: phosphoinositide 3-kinase
PLEKHM1: pleckstrin homology domain containing protein family member 1
PPAR: peroxisome proliferator-activated receptor
PtdIns: phosphatidylinositol
PTPN2: tyrosine-protein phosphatase non-receptor type 2
ROS: reactive oxygen species
SNAP29: synaptosome-associated protein 29
SNARE: soluble N-ethylmaleimide-sensitive factor attachment protein receptor
STX: syntaxin
TNF: tumour necrosis factor
TOR: target of rapamycin
UC: ulcerative colitis
ULK: UNC-51-like kinase
UVRAG: UV radiation resistance-associated gene product
VAMP: vesicle-associated membrane protein
VPS: vacuolar protein sorting-associated gene product
WIPI: WD-repeat protein interacting with phosphoinositides
XBP-1: X-box-binding protein-1
